# Medical high-entropy alloy: Outstanding mechanical properties and superb biological compatibility

**DOI:** 10.3389/fbioe.2022.952536

**Published:** 2022-08-11

**Authors:** Changxi Liu, Chengliang Yang, Jia Liu, Yujin Tang, Zhengjie Lin, Long Li, Hai Liang, Weijie Lu, Liqiang Wang

**Affiliations:** ^1^ State Key Laboratory of Metal Matrix Composites, School of Material Science and Engineering, Shanghai Jiao Tong University, Shanghai, China; ^2^ National Center for Translational Medicine, Shanghai Jiao Tong University, Shanghai, China; ^3^ Department of Orthopaedics, Affiliated Hospital of Youjiang Medical University for Nationalities, Guangxi Key Laboratory of Basic and Translational Research of Bone and Joint Degenerative Diseases, Baise, China; ^4^ 3D Printing Clinical Translational and Regenerative Medicine Center, Shenzhen Shekou People’s Hospital, Shenzhen, China; ^5^ Department of Stomatology, Shenzhen Shekou People’s Hospital, Shenzhen, China

**Keywords:** bio-heas, mechanical properties, corrosion resistance, cytocompatibility, friction resistance

## Abstract

Medical metal implants are required to have excellent mechanical properties and high biocompatibility to handle the complex human environment, which is a challenge that has always existed for traditional medical metal materials. Compared to traditional medical alloys, high entropy alloys (HEAs) have a higher design freedom to allow them to carry more medical abilities to suit the human service environment, such as low elastic modulus, high biocompatible elements, potential shape memory capability. In recent years, many studies have pointed out that bio-HEAs, as an emerging medical alloy, has reached or even surpassed traditional medical alloys in various medical properties. In this review, we summarized the recent reports on novel bio-HEAs for medical implants and divide them into two groups according the properties, namely mechanical properties and biocompatibility. These new bio-HEAs are considered hallmarks of a historic shift representative of a new medical revolution.

## Introduction

Currently, numerous biomaterials, including polymer materials, composite materials, and metal materials, have been developed for disease visualization, drug toxicity assessment and detection, tissue repair and substitution (Gaharwar et al., 2020; Eliaz, 2019; Fenton et al., 2018; Qu et al., 2019; Mitrousis et al., 2018; Wang et al., 2021a; Liu et al., 2022). With excellent mechanical properties and good corrosion resistance, metal medical implants undertake the function of repairing and replacing human diseased tissues and organs, which are widely used in artificial heart valves, bone implants and scaffolds, and tooth repair and replacement (Eivazzadeh-Keihan et al., 2020; Sharma et al., 2020; Wang et al., 2022a; Guo et al., 2022; Mao et al., 2022; Qiao et al., 2022) Figure 1A illustrates the several stages of metal implant development and improvement. The earliest metal implant material is 304 stainless steel (Verran and Whitehead, 2005), which is used in artificial joints. Stainless steel material has high strength but also has a high elastic modulus (193 GPa), which is much higher than human bones 10–40 GPa). Such mismatched elastic moduli will prevent the load from smoothly transferring from the implant to the surrounding bone tissue, resulting in a stress shielding phenomenon when the implant is implanted into human bone (Mi et al., 2007). The stress shielding phenomenon can lead to the degeneration and atrophy of the bone tissue and eventually cause the implant to loosen and even fail, which does not meet the requirements of long-term service. Subsequently, compared with other metal materials, titanium and titanium alloys have the characteristics of high specific strength, strong corrosion resistance and good biocompatibility and have become preferred materials for bone tissue repair and replacement (Wang et al., 2021b; Wang et al., 2022b; Cui et al., 2022; Lv et al., 2022; Zhang et al., 2022). For instance, Wang developed a Ti-35Nb-2Ta-3Zr (wt%) alloy with a low Young’s modulus of approximately 48 GPa (Wang et al., 2017).

**FIGURE 1:**
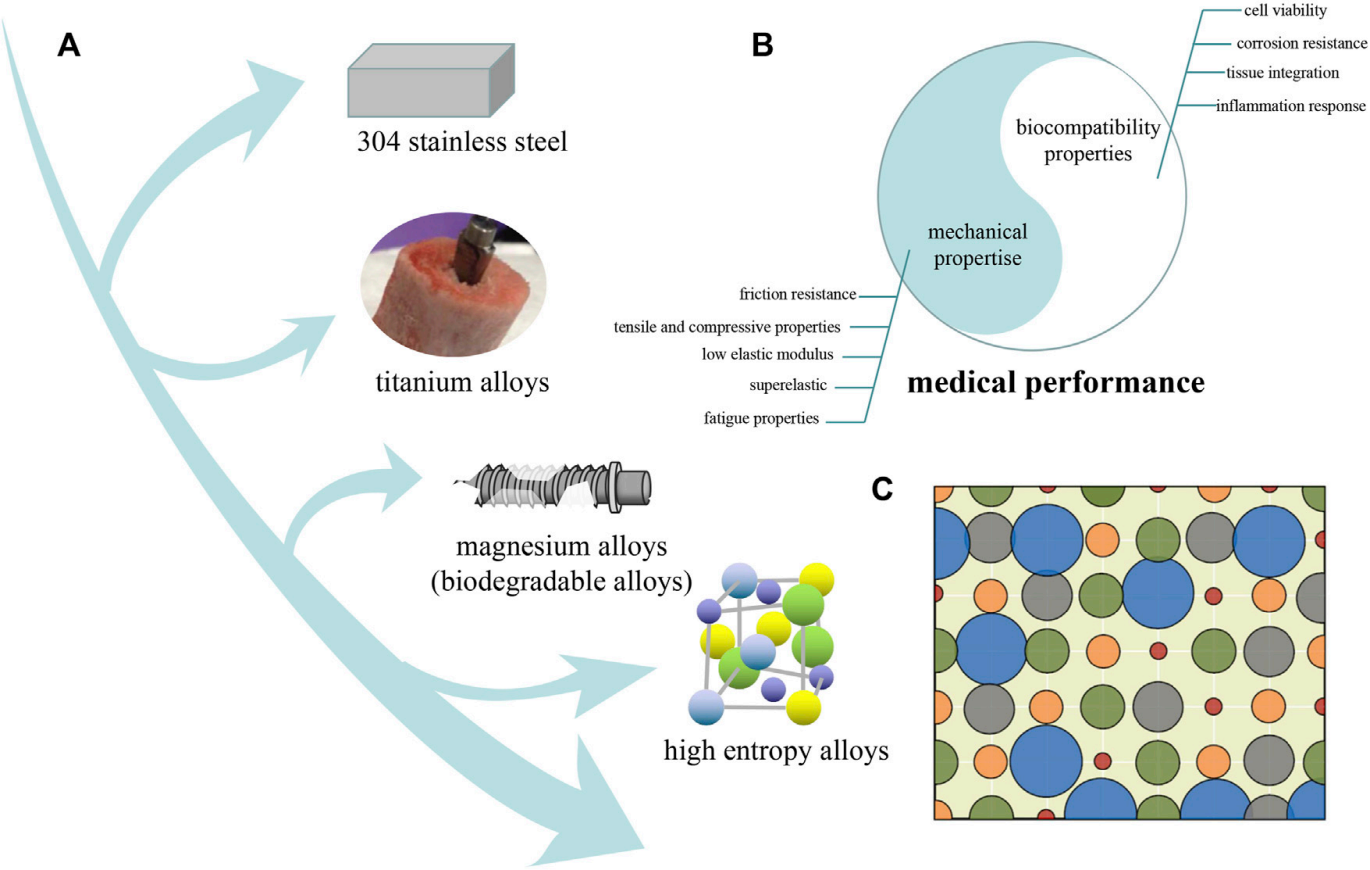
**(A)** Schematic showing several different medical metal implant development sequences. **(B)** Mechanical properties and biocompatibility constitute the performance evaluation criteria for medical metal implants. **(C)** Schematic diagram showing the elemental arrangement model of HEA (the circles in different colors and varying sizes represent the mixed atoms), in this case, the configurational entropy is determined not only by chemical composition but also by atomic size (Ye et al., 2016).

High-entropy alloys have received a great amount of attention in recent years because of their unique composition (five or more metal elements) and homogeneous microstructure (Cantor et al., 2004; Hemphill et al., 2012; Gludovatz et al., 2014; Yu et al., 2014; Ye et al., 2015a; Xia et al., 2015; Miracle and Senkov, 2017). Compared to conventional alloys with relatively simple compositions, HEAs have two significant features: 1) enormous room for performance optimization and improvement derives from multielemental combinations, which ensure that HEAs have a variety of ingredients and complex microstructures, and 2) the various elements mixed will exhibit properties that are not possessed by any pure metal element, which provides HEAs with new properties (Tsai and Yeh, 2014; Lu et al., 2015; George et al., 2019).

With the excellent performance of HEAs, a good approach is to put HEAs into the medical field to explore the potential of HEAs as medical implants. Furthermore, HEAs represented by Ti, Ta, Nb, Zr, and Hf systems have good application and development potential in medical implants, as shown in Figure 1B (Raducanu et al., 2011; Wang and Xu, 2017; Tüten et al., 2019; Yang et al., 2020). For instance, bio-HEAs could be designed to possess high strength following rational guidance due to their complex elemental composition and richness of design (Wang et al., 2020a; Su et al., 2022; Xu et al., 2022). On the other hand, bio-HEAs are designed to have low toxicity because the elements in bio-HEAs have excellent biocompatibility (Nagase et al., 2020). This review aimed to discuss recent advances in bio-HEA mechanical properties and biocompatibility.

## High entropy alloy concept

The current mainstream concept is that the high entropy alloy should contain 5–13 main elements, and the mole fraction of each element should be between 5 and 35 at% (Cantor et al., 2004; Yeh et al., 2004). The term “high-entropy alloy” is defined because the relationship of individual atoms can be modelled as an ideal solution, as illustrated in Figure 1C.

The atomic radius difference range (*δ*) (1) is used to describe the radius relative radius of each element (‾r is the average atomic radius and r_i_ is the atomic radius of element i). It claims that only solid solutions are formed when the *δ* value is lower than 4% (Zhang and Lv, 2008).

$$\delta =100\sqrt{\sum c_{i}\left(1-\frac{r_{i}}{\bar{r}}\right)}$$ (1)

Configuration entropy (ΔS_am_) 2) is used to describe the mixing entropy of alloys, which high entropy alloys require the ΔS_mix_ value are higher than 11 J/mol K, where c_i_ is the molar fraction of the *i*th element, R is the constant (8.314 J/mol K), and n is the total number of constituent elements.

The enthalpy of mixing (ΔH_am_) 3) range of the HEA is a key parameter, which requires between −11.6 and 3.2 kJ/mol, where ΔH_ij_ is the binary enthalpy of elements i and j (Zhang and Lv, 2008).

$$\Delta S_{\mathrm{am}}=-R\sum c_{i}\text{ln}c_{i}$$ (2)

$$\mathrm{\Delta Ham}=\sum c_{j}\mathrm{\Delta Hij}$$ (3)

The parameter *O* 4) involves ΔS_am_ and ΔH_am_, which can predict the composition of the final HEA phase, where T_top_ is the melting temperature calculated using 5. Usually, only solid solutions are formed when *Ω* > 1.1 and *δ* < 3.6%, and only solid solutions and intermetallic compounds are formed when 1.1 < *O* < 10 and 3.6% < *d* < 6.6%; furthermore, only solid solutions are formed when *Ω* > 10 (Yang and Zhang, 2012).

$$\Omega =T_{\mathrm{top}}\Delta S_{\mathrm{am}}/\left|\mathrm{\Delta Ham}\right|\;$$ (4)

$$T_{\mathrm{top}}=\sum c_{i}T_{\mathrm{top}\;i}$$ (5)

The difference in electronegativity Δχ 6) is also used as the criterion for judging whether a single solid solution can be formed, where *χ* is the average electronegativity and χ_i_ is the electronegativity of element i. Only solid solutions are formed when the components of the alloy elements are between 3 and 6%.

$$\Delta \chi =100\sqrt{\sum c_{i}\left(1-\frac{\chi_{i}}{\chi }\right)2}$$ (6)

Although there may be some differences in the above criteria in the current research on high-entropy alloys, these criteria are useful for predicting the solid phase and composition selection and determination of the atomic fraction ratio for bio-HEAs (Takeuchi et al., 2013; Ye et al., 2015b).

## HEAs design and numerical simulation

Compared with traditional alloys, bio-HEAs have a high freedom in element design, which also means more factors need to be considered. Multi-element of bio-HEAs not only induce strong chemical fluctuation to random phase, but also have important effects on the stacking fault energy, second phase and dislocation core structure. In fact, numerical simulations could reflect the bio-HEAs properties independent of material test, which is very beneficial for guiding the design of alloys with high freedom such as bio-HEAs (Yin et al., 2020; Xu et al., 2021). In recent years, Monte Carlo (Zhou et al., 2021), molecular dynamics (Jian et al., 2020; Li et al., 2020), first-principles calculations (Rao et al., 2019), and deep learning (Kostiuchenko et al., 2019) is widely applied in modeling and prediction of bio-HEAs.

Lee (Lee et al., 2020) predicted the Young’s modulus (E), bulk modulus (K) and shear modulus (G) of the material by First-principles calculations, showing excellent agreement with the experimental values. Moreover, Yao (Yao et al., 2016) established phase diagrams of NbTaTiV, NbTaVW, and NbTaTiVW through CALPHAD modeling, which predicted the state of NbTaV(TiW) in different temperature ranges. Liu (Liu et al., 2021) used Monte Carlo to predict order-disorder transitions caused by W and Nb. By comparing with experiments, the simulation results provide insight into the role of chemical ordering in the strength and ductility of HEAs.

In fact, HEAs with so many element combinations means huge data and this is a perfect example of deep learning. With the rapid development of computer science, deep learning is becoming more and more accurate for analyzing data patterns and predicting development from samples (Lecun et al., 2015; Guo et al., 2016). For the design of HEAs, the optimal HEA potential element ratio could be output by a deep learning network trained on HEA experimental data (Yan et al., 2021). For instance, wen (Wen et al., 2021a) collected experimental data on AlCoCrFeNi, CoCrFeNiMn, HfNbTaTiZr, and MoNbTaWV, and trained a deep learning network for these types of HEAs to predict the potential of solid solution strengthening. In the future, deep learning might play a guiding role in the design and prediction of potential properties of HEAs.

## Mechanical properties

Compared with traditional medical metal implants, HEAs have excellent mechanical properties (Picak et al., 2021; Wei et al., 2022a). The strength, ductility, elastic modulus and fatigue properties of bio-HEAs should be considered. The excellent mechanical properties of these medical high-entropy alloys are inextricably connected with their microstructures. In fact, the microstructures of HEAs are numerous and complex, and the final microstructure is not the same even for HEAs with the same elements but different ratios, furthermore, heat treatment and thermal deformation also affect the structure (Dirras et al., 2016; Zhang et al., 2018; Li et al., 2019). It is critical to obtain an overall view and summarize the properties of bio-HEAs at this stage, as well as to determine the design ideas of future bio-HEAs.

### Tensile and compressive properties

**FIGURE 2:**
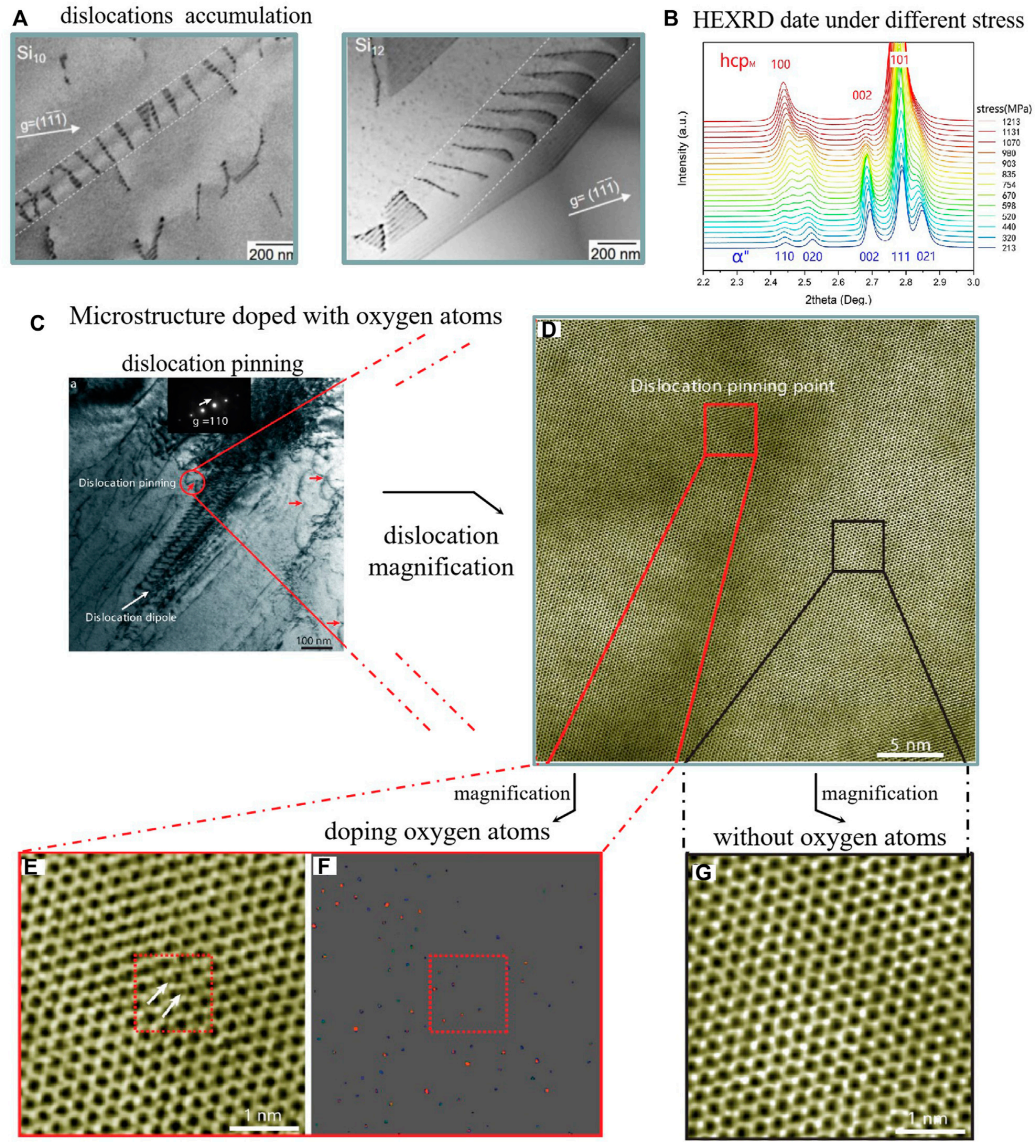
**(A)** Two-beam BF images show the frequently observed accumulation of dislocations on the {111}-type FCC slip planes in Co_22_Cr_22_Fe_22_Ni_22_Si_10_ and Co_10_Cr_10_Fe_10_Ni_10_Mn_10_Si_10_HEA (Wei et al., 2022b). **(B)** the (100) peak of hcpM could be found when the stress was over 520 MPa **(C)** Dislocations in the 8% strained O-2 HEA, imaged under {1a11}-type diffraction conditions (Wang et al., 2020b). **(D)** The dislocation pinning point (red circle) in **(C)** was chosen for further STEM characterization. **(E)** Aberration-corrected STEM-ABF images of the local atomic structure of pinning sites. White arrows point to the pillars of oxygen atoms. (**F)** STEM-HADDF also indicates ordered oxygen complexes near the dislocation pinning point **(G)** Aberration-corrected STEM-ABF images far from the pinning point (Lei et al., 2018).

Tensile and compression tests are some of the most intuitive criteria to detect the mechanical properties of materials, which can obtain a series of material performance data, such as yield strength, breaking strength, and elongation, from stress–strain diagrams. Furthermore, many HEAs with excellent mechanical properties have been developed in recent years, which yield strength exceeding 1000 MPa and the elastic modulus lower than 70 GPa These HEAs often have high tensile strength and excellent elongation data, achieving simultaneous improvement of material strength and plasticity. Wei (Wei et al., 2022b) replaced part of the metal elements in HEAs with the metalloid element Si, in which the metalloid element is between metals and nonmetals, and it is easy to induce complex subnanometre-scale structures in the substrate. Figure 2A illustrates the accumulation of dislocations on the {111}-type FCC slip planes. The mechanical properties of -Si HEAs are improved due to these defects, which is consistent with the results of first-principles calculations and Monte Carlo simulations. It shows the elevation of ductility simultaneously with strength in macroscopic performance. Su (Su et al., 2019) designed a hierarchical microstructural for HEA by introducing grains and textures with different size gradients and substructures, which enhanced transformation-induced plasticity (TRIP) and twinning-induced plasticity (TWIP) effects. The material exhibits bimodal microstructures, which were produced consisting of nanograins (∼50 nm) in the vicinity of shear bands and recovered parent grains (10–35 μm) with preexisting nanotwins after tempering. Compared with the 95% recrystallized specimen’s yield strength of 555 MPa, the HEA yield strength of the bimodal microstructures is increased to 1.3 GPa.

The equiatomic HfNbTaTiZr achieves a tensile yield strength of 974 MPa and has an elongation of 20%. Furthermore, there are numerous dislocations with restricted movement at grain boundaries in HfNbTaTiZr, due to grain refinement (Juan et al., 2016). The O element doped TiZrHfNb exhibited a yield strength of 1,300 MPa and an elongation of 30% in the room temperature tensile test. The strong ordered oxygen complexes in Figures 2C–G are the key reason to achieve such performance. The strength improvement is due to the O solid solution strengthening, more interestingly, the plasticity improvement is due to O changing the plastic deformation mode from plane slip to wave slip, which is different from conventional alloys (Lei et al., 2018). This study shows that the presence of interstitial oxygen elements in a nano-ordered manner could successfully overcome the strength–ductility trade-off.

Chen (Chen et al., 2022a) found that the WNbMoTaZr HEA has a significant increase in strength and toughness with increasing Zr content; the yield strength in the compression test is 1,223 ± 20.1 MPa, and the fracture strain is 6.4 ± 0.66%. In addition, TiZrNbTa doped with N also achieved high strength and high toughness of the material. The yield strength and fracture strain of the tensile test reached 1,196 ± 8 MPa and 17.5 ± 0.3%, respectively (Wang et al., 2022c). The introduction of N in the original matrix resulted in dendritic structures and simultaneously led to dislocation pinning and reduce diffusion rate.

**FIGURE 3:**
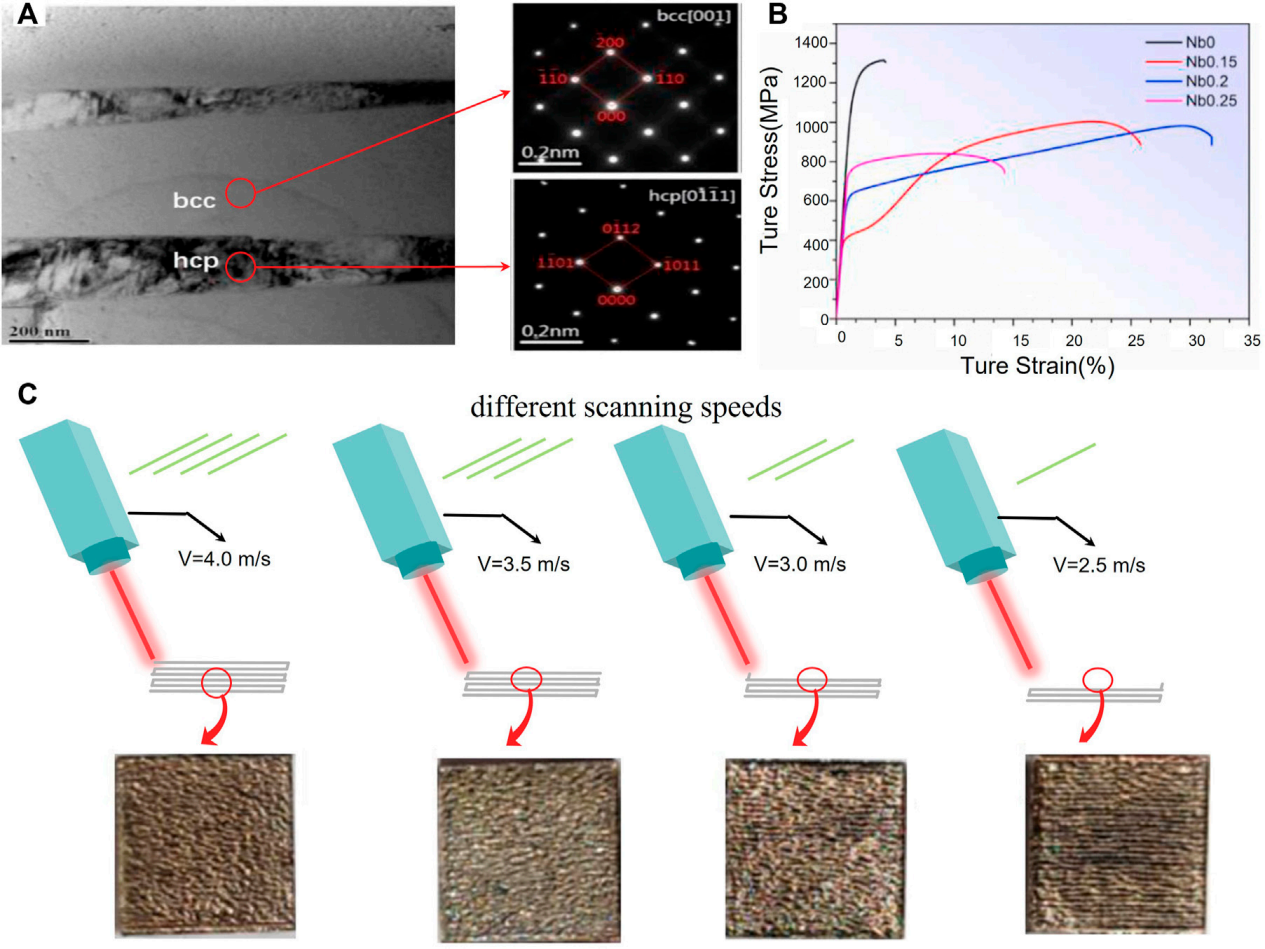
**(A)** TEM bright-field images of the co-existed BCC and HCP phase (Wen et al., 2021b). **(B)** Selected area diffraction show the extra twinning spots **(C)** Four different scanning speeds at 4.0 m/s, 3.5 m/s, 3.0 m/s, 2.5 m/s for the SLM (Xiao et al., 2022).

In recent years, some studies on the microstructure and mechanism of bio-HEAs may provide theoretical support for high-strength mechanical properties. Lee believes that unlike traditional body-centered cubic (BCC) structure metals and dilute alloys, in which the strain strengthening depends on screw dislocation, plastic flows in HEAs mainly contribute to edge dislocation (Lee et al., 2021). Furthermore, TWIP and TRIP are still the main methods to enhance the mechanical properties of bio-HEAs. During the tensile process, TWIP and TRIP occur sequentially in the *ß* phase. Ti_16_Zr_35_Hf_35_Ta_14_ was found to exhibit a new stress-induced martensitic transformation (SIMT) α”-to-hcpM by *in situ* high energy X-ray diffraction (HEXRD), as shown in Figure 2B. The peak of hcpM starts to appear when the stress is 520 MPa, and the peak of α” gradually weakens and finally disappears when the stress is 800 MPa. In addition, SIMT improves the yield strength-ductility of Ti16Zr35Hf35Ta14 ^72^. Wen (Wen et al., 2021b) found that the Nb content in HfNbTa_0.2_TiZr HEA affects the stability of the BCC phase. With the decrease in Nb content, the martensite tends to transform from the BCC structure to the HCP structure, as shown in Figure 3A. A large number of twins are observed during the transition, which may be due to the lower SFE of the HCP structure, and the material finally exhibits a dual increase in strength and plasticity, as shown in Figure 3B. In addition, the fine precipitates in Hf_20_Nb_10_Ti_35_Zr_35_ formed by ageing cooperate with TWIP and TRIP and improve the mechanical properties of the material (Su et al., 2022).

Additive manufacturing (AM) is a new type of manufacturing method that has a great impact on medical implant fabrication and its ability to produce complex, porous configurations and structure-specific implants (Herzog et al., 2016; Bourell et al., 2017). Additively manufactured porous materials fabricated by AM could well mimic the skeletal environment in which cells grow (Hafeez et al., 2020; Zhang et al., 2021a). Compared with the HEAs prepared by cold crucible suspension, the microstructure fabricated by AM may be different due to different cooling rates during fabrication; furthermore, the final mechanical properties of the material are also different. Defects caused by AM are an important factor that affects the performance of bio-HEAs because the elements used in bio-HEAs not only have high melting points but also have a wide range of melting points between different elements. Zhang (Zhang et al., 2021b) found that a mixed powder of NbMoTa has a high defect rate after fabrication. Consequently, the printed material has higher formability and strength for SLM after adding Ti and Ni elements. Compared with NbMoTa, the NbMoTaTi_0.5_Ni_0.5_ HEA has a large amount of extended dislocation at the grain boundary, which strengthens the grain boundary of the crystal. Xiao (Xiao et al., 2022) studied the effect of selected laser melting (SLM) on the microstructure and mechanical properties of WMoTaNbTi HEAs. Several different scan speeds, including 4.0 m/s, 3.5 m/s, 3.0 m/s and 2.5 m/s, were collected and are illustrated in Figure 3C. The material exhibited the highest compressive strength of 1,312 MPa and exhibited good local ductility when the scanning speed was 2.5 m/s.

### Low elastic modulus HEAs

Reducing the elastic modulus of metal implants to match human bone to prevent potential stress shielding risks is an important goal for medical metal implants. TiZrNb, Ti_40_Zr_40_Nb_20_, Ti_45_Zr_35_Nb_20_, Ti_45_Zr_45_Nb_10_, and Ti_50_Zr_40_Nb_10_ all exhibit low elastic moduli (Hu et al., 2022). In particular, the TiZrNb HEA, at room temperature, is composed of dendritic crystals with a single BCC, whose elastic moduli range from 73 ± 3 GPa to 52 ± 2 GPa and are very close to the elastic moduli of large human bones. Schönecker (Schönecker et al., 2022) proposed a new idea for reducing the elastic modulus of TiZrNbMoTa. Considering the service conditions of the bones, including walking, running, and climbing, the load direction of the leg is along the long-bone (longitudinal direction). For this unidirectional loading situation, the anisotropy of the material is used to reduce the elastic modulus. Single crystals and textured polycrystals have lower elastic moduli than isotropic materials in a certain direction.

In fact, the elastic modulus of a material is affected by the chemical bond, crystal structure, chemical composition, etc. The bio-HEAs could form numerous types according to different ratios and selected elements. Such a large number of samples undoubtedly provides sufficient samples for machine learning to calculate and predict the final performance of materials. In recent years, with the rapid development of computer science, machine learning, as a product of the development of computer science, has been widely used in the calculation of big data (Dove et al., 2017; Wei et al., 2019). Roy (Roy et al., 2020) used the gradient boost for a regression model to predict 26 high-entropy alloys with different compositions, in which the deviation from the test value of the sample did not exceed 20%, and the elastic modulus of TaTiZr was predicted to be 98.33 GPa. Compared with blindly arranging and combining element types, machine learning can provide a relatively clear path in designing materials.

### Superelastic HEA

In 1963, the Naval Ordnance Laboratory discovered superelasticity in TiNi alloys with nearly equiatomic proportions (Buehler et al., 1963). To date, supereelastic metals have been widely used in aerospace, marine and cable communications (Hoh et al., 2009). In medical metal implants, bone scaffolds fabricated by superelastic materials have better flexibility and better fit the complex structure inside the human body.

**FIGURE 4:**
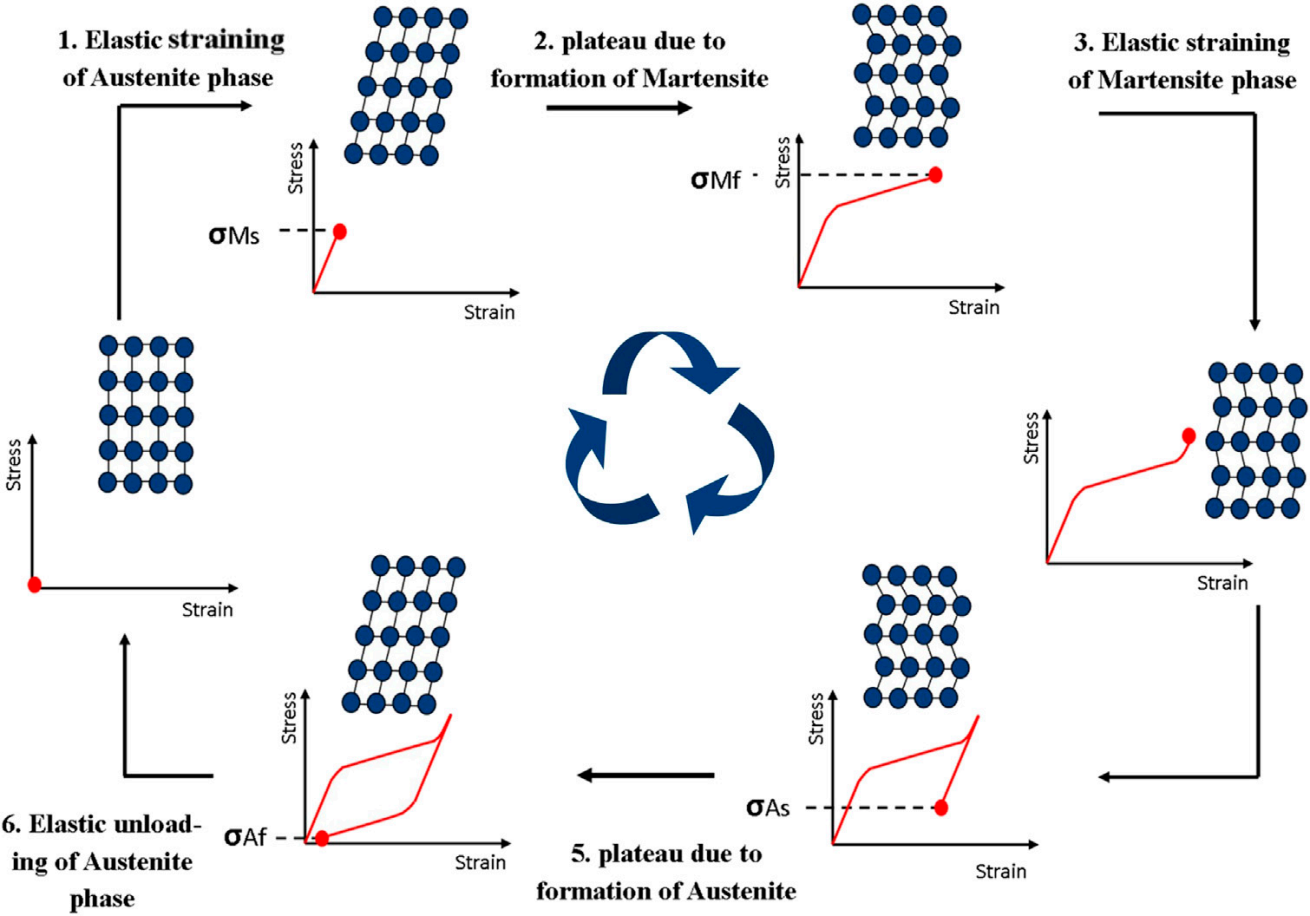
The schematic diagram for mental superelasticity by martensitic transformation (Ramezannejad et al., 2019).

SIMT could improve the mechanical properties of HEAs; for instance, TRIP could improve the plasticity of HEAs. In addition, SIMT is a prerequisite for superelasticity effects and shape memory of mental materials. The *ß* phase in the superelastic alloy transforms into orthorhombic α″ martensite when loaded up to a certain critical stress (*s*_SIM_). The α″ martensite grows through several variants that yield to the maximum strain along the loading direction. These repeatedly loaded trajectories exhibit a cyclic shape in the stress–strain curve in Figure 4 (Ramezannejad et al., 2019). Upon unloading, α″ martensite is able to totally transform back to *ß* under the ideal scenario.

Some bio-HEAs have also been shown to have superelastic properties. Peltier (Peltier et al., 2021) pointed out that for (TiHfZr)_74_(NbTa)_26_, its superelasticity originates from the transition of β↔α” when deformation occurs. The superelastic temperature range is 40–200°C, which is compatible with the human service environment temperature. Furthermore, Wang (Wang et al., 2019) utilized *in situ* XRD to characterize the superelastic behavior of TiZrHfAlNb and found β↔α” during unloading in experiments on uniaxial tension. In addition, the plastic deformation is also recovered during this process. The discovery of these superelastic bio-HEAs will encourage more scholarly interest and attention.

### Fatigue properties of HEA

Fatigue fracture often occurs when the material is under the action of alternating loads for a long time, and the material is broken by a stress lower than the breaking strength. Fatigue fracture is due to the initiation of internal cracks, and the cracks gradually propagate under alternating loads until failure. Metal medical implants are often under alternating loads during service. For instance, artificial teeth undergo hundreds of times of chewing every day. These chewing movements can be regarded as materials that are under the alternating stress environment.

HfNbTaTiZr exhibits good performance in fatigue tests. The maximum stress required to exceed the yield stress causes fatigue failure of HfNbTaTiZr even in the high cycle fatigue regime (Guennec et al., 2018a). In the microstructure, the fatigue strength of the material is affected by the mobility of dislocations, which increases with mobility. This feature has also been observed in other alloys (Mughrabi and Wüthrich, 1976; Magnin and Driver, 1979; Guiu et al., 1982; Guennec et al., 2015). In the low-cycle regime of HfNbTaTiZr, the resistance to fatigue is through the accumulation of dislocations at the crack tip, which can lead to the closure of the crack (Chen et al., 2022b).

In fact, currently, more fatigue research on HEAs focuses on FeCoNi systems (Hemphill et al., 2012; Tang et al., 2015; Thurston et al., 2017; Guennec et al., 2018b), and research on medical high-entropy alloys is still lacking. To date, medical high-entropy alloys are mainly aimed at strength and toughness, as well as low modulus. Fatigue performance is an important medical indicator; therefore, fatigue behaviour investigations on bio-HEAs are suggested to obtain a wider range of bio-HEAs with good fatigue performance.

## Biocompatibility properties of Bio-HEAs

Compared with the HEA materials used in the manufacturing industry and aerospace industry, which require metals to have excellent mechanical properties, bio-HEAs not only require good mechanical properties but also require additional materials with excellent biocompatibility properties. Furthermore, the purpose of testing biocompatibility is to explore the potential biological risks when medical high-entropy alloys are used as implants. In recent years, many scholars have extensively studied the biocompatibility of HEAs and found many bio-HEAs with excellent biocompatibility. The biocompatibility of bio-HEAs, including cytocompatibility, corrosion resistance, friction resistance, and bio-HEAs with these three excellent properties, is summarized.

### Cytocompatibility

The cell viability experiment is the most intuitive test to analyze the biocompatibility of bio-HEAs. The purpose of the cell viability experiment was to simulate cell growth and differentiation and to observe whether the cells still have normal functions on the implant surface. For instance, osteoblasts are often cultured on the implant surface to observe osteoblast division, differentiation, and the final mineral deposition quality (McBeth et al., 2017). At present, many studies have shown that medical high-entropy alloys exhibit high cell viability and provide a good environment for cell work.

Todai (Todai et al., 2017) found that TiNbTaZrMo HEA exhibited excellent biocompatibility, and the osteoblast activity attached to the surface was closely related to the microstructure of HEA. SUS316L, CP-Ti, and TiNbTaZrMo HEAs in the as-cast and annealed samples were tested in total, and it was pointed out that the osteoblast density of TiNbTaZrMo in the as-cast and annealed samples was higher than that of SUS316L and CP-Ti. In addition, TiNbTaZrMo in the annealed sample exhibited the highest cell density and was superior to SUS316L in cell size and cell spreading, which are important for cell migration and protein synthesis. Furthermore, this study also pointed out that the annealed TiNbTaZrMo has better cytocompatibility due to the redistribution and grain growth of the annealed grains. Shittu (Shittu et al., 2020) showed that MoNbTaTiZr HEA not only has good mechanical properties, in which the elastic modulus is 30% lower than that of SS304 but also has good cytocompatibility. Stem cells were cultured onto MoNbTaTiZr and tissue culture polystyrene (TCPS) surfaces, and fluorescence microscopy was used to show cellular coverage. The cell coverage of MoNbTaTiZr and TCPS reached 89 and 100%, respectively. Furthermore, numerous long cytoplasmic extensions forming a network in contact with adjacent cells were observed on the bio-HEA surface, indicating that the MoNbTaTiZr surface supports cell attachment by filopodia extensions and provides strong support for the growth of cells (Hasan et al., 2017).

**FIGURE 5:**
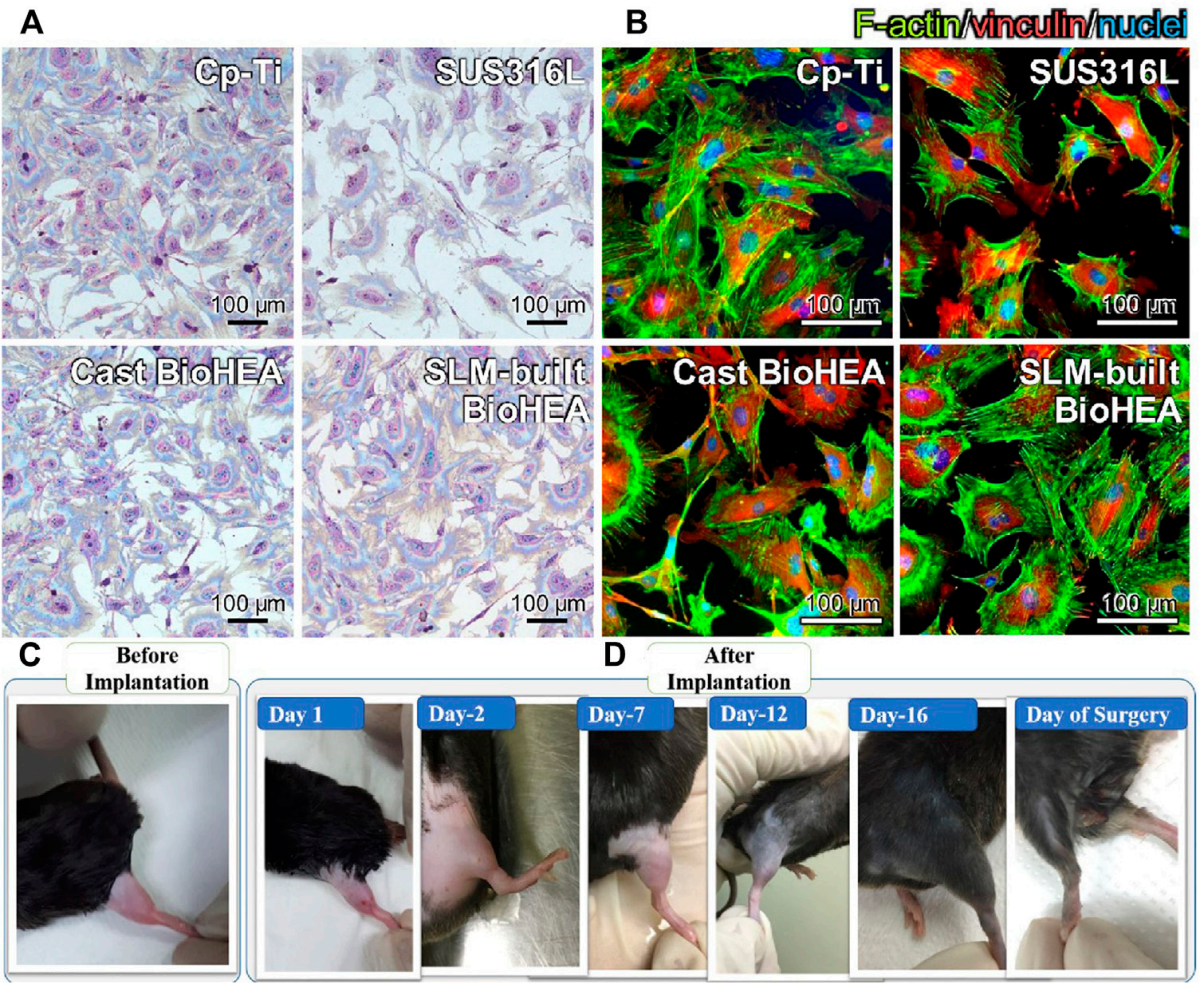
**(A)** Giemsa staining images of osteoblasts cultured on the surface of SLM-built BioHEA and CP-Ti, SS316L, and cast BioHEA counterpar (Ishimoto et al., 2021)t. **(B)** Fluorescent images of osteoblast adhesion on SLM-built bio-HEA, CP-Ti, SS316L, and cast bio-HEA (Akmal et al., 2021). **(C,D)** Visual evidence of mice thigh before and d after implantation (MoTa)0.2NbTiZr alloy (Akmal et al., 2021).

The TiNbTaZrMo fabricated by SLM not only has superior mechanical properties but also has great biocompatibility. Giemsa staining images showed that the cell growth density of bio-HEAs fabricated by SLM was the same as that of CP-Ti and much higher than that of SS316L, as shown in Figure 5A. Furthermore, fluorescent images demonstrate the cell cytoskeletal components and focal adhesions of osteoblasts adhered to the specimens in Figure 5B. The cells exhibited a uniform distribution on the bio-HEA surface, which had an obvious advantage of cell spreading. Such great mechanical and biological properties of bio-HEAs are due to the rapid solidification in the SLM fabrication process, which can effectively inhibit the segregation of components (Ishimoto et al., 2021).

Animal models are an effective approach to evaluate the service status of materials in the *in vivo* environment. Akmal (Akmal et al., 2021) demonstrated (MoTa)_x_NbTiZr implantation inside a mouse thigh and counted the changes in the mouse thigh over 16 days, as shown in Figures 5C,D. The mouse thigh was inflamed after implantation, and after Day 7, the inflammation subsided without abnormal neurobehaviour. However, host response experiments including inflammatory response, osteoinductive and bioactive behavior still lack additional investigation, and *in vitro* experiments should be further discussed.

### Corrosion resistance

Metal implants may have a potential risk of corrosion in the human body, which may lead to a decrease in implant performance and failure. Bio-HEAs have shown good potential in corrosion resistance properties, and research on corrosion resistance will broaden bio-HEA applications in medical materials.

**FIGURE 6:**
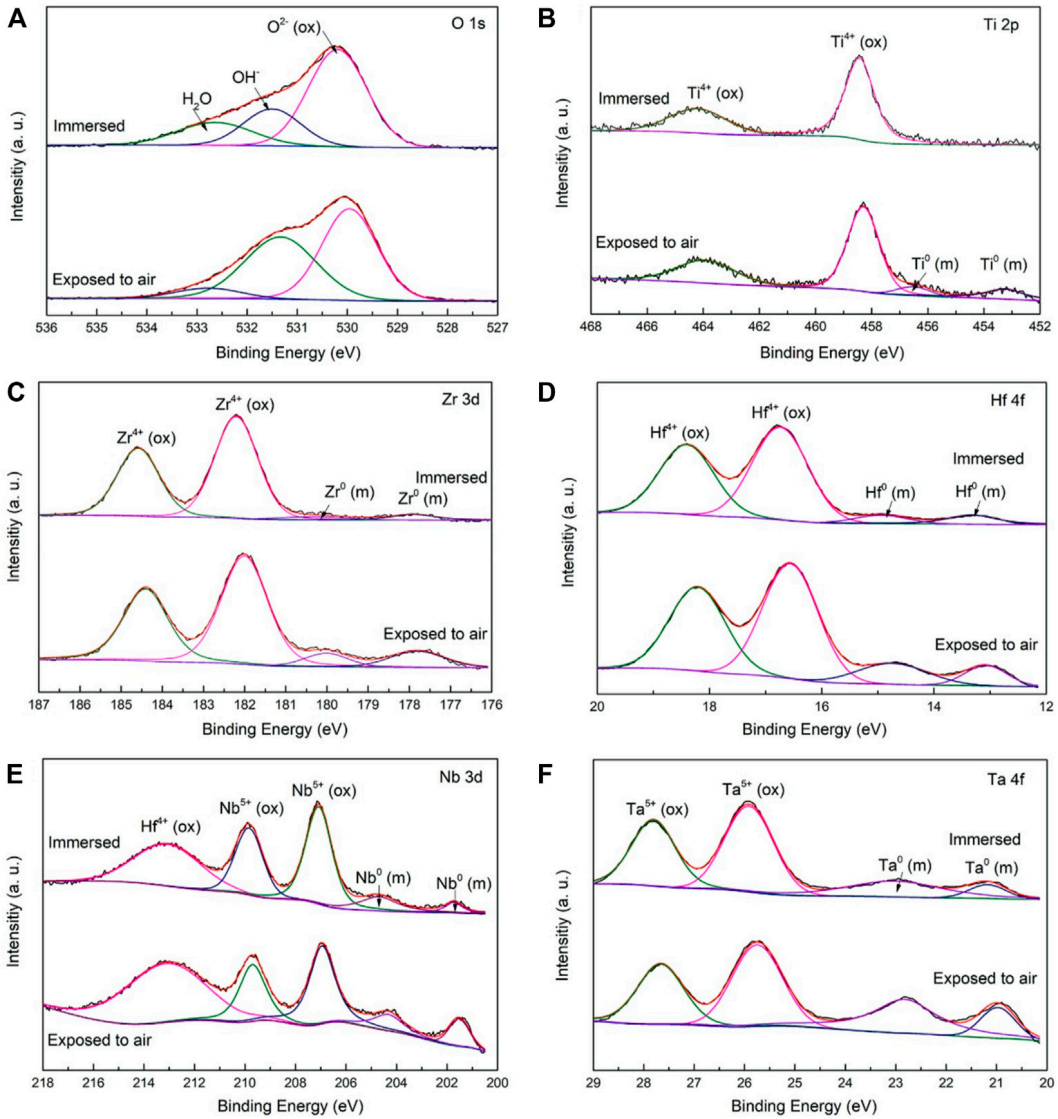
XPS spectra of the surface films formed on the TiZrHfNbTa [**(A)** O 1s, **(B)** Ti 2p, **(C)** Zr 3d, **(D)** Hf 4f, **(E)** Nb 3d, and **(F)** Ta 4f], which exposed to air and after the 7-days immersion in the Hank’s solution at 310 K^33^.

In a corrosive environment, bio-HEAs are oxidized, and a passive oxide film grows on the surface. The density of the oxide film is a key factor in preventing further corrosion and avoiding material failure. Yang (Yang et al., 2020) pointed out that the corrosion rate of TiZrHfNbTa HEA is 10–4 mm/year under an environment of a low passive current density of approximately 10–2 A/m2, comparable to the traditional Ti6Al4V alloy. Through X-ray photoelectron spectroscopy (XPS) tests, it was found that TiO2, ZrO2, HfO2, and Ta2O5 were formed during the corrosion process, which played an important role in resisting corrosion, as shown in Figures 6A–F. Furthermore, Wang (Wang et al., 2022d) replaced the element Ta in TiZrHfNbTa with element Fe to test the effect of different volume fractions of Fe elements (0, 0.25, 0.5, 0.75, 1, 1.5, 2) on the material corrosion resistance. It should be noted that the corrosion potential first decreased and then increased with increasing Fe content. Fe_0.5_ exhibited the best corrosion resistance, and no corrosion pits were observed after polarization. Hua pointed out that TiZrNbTaMo also has great corrosion resistance. TiZrNbTaMo has better corrosion potential than traditional Ti6Al4V, which means that the passivation film produced by TiZrNbTaMo has higher stability (Hua et al., 2021).

### Friction resistance performance

**FIGURE 7:**
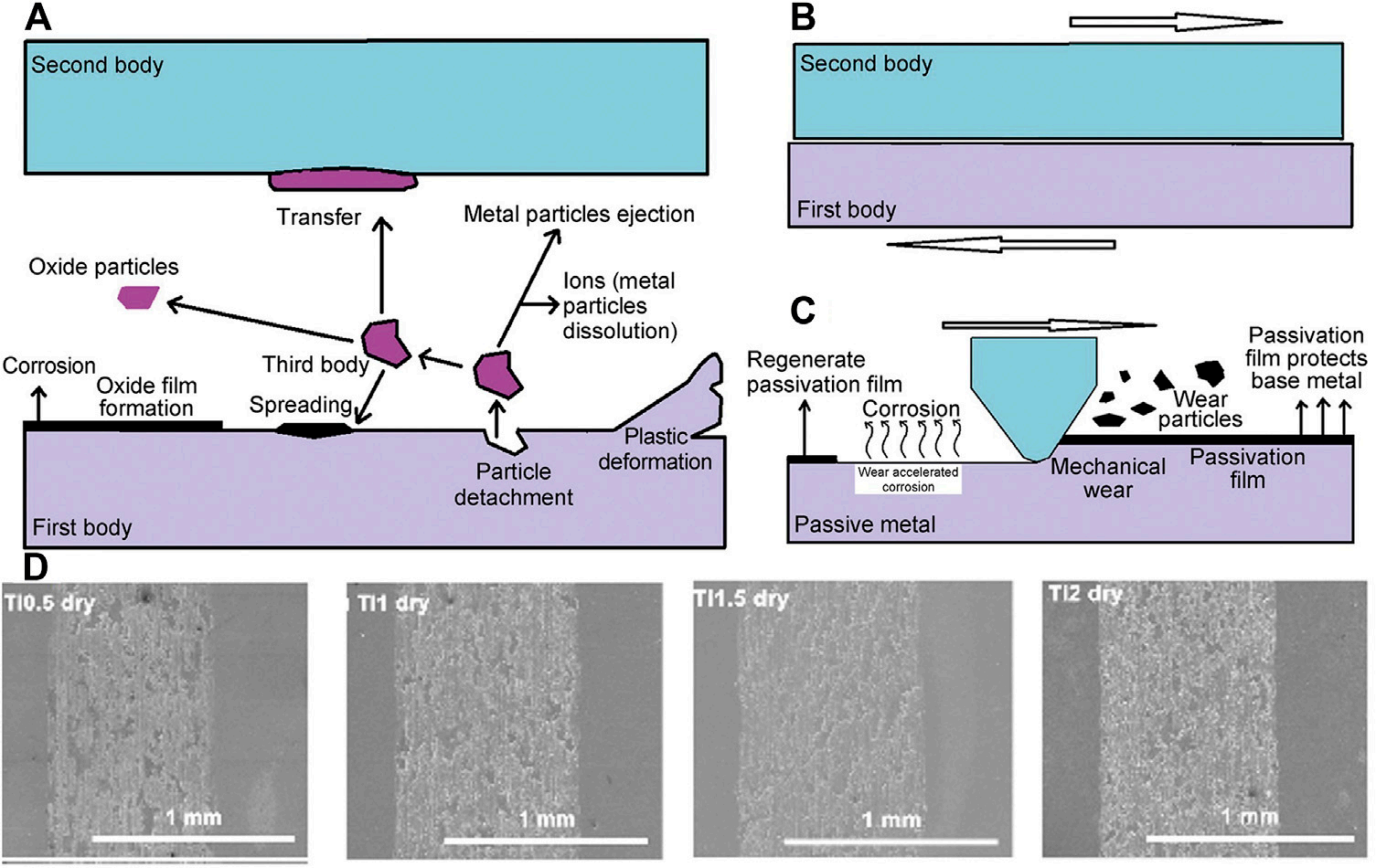
Schematic diagram of friction corrosion mechanism of implant surface: **(A)** three-body wear; **(B)** two-body wear; **(C)** removal process of the passivation film (Li et al., 2021). **(D)** SEM images of wear scars of the Ti_0.5_ZrNbTaMo, TiZrNbTaMo, Ti_1.5_ZrNbTaMo, and Ti_2_ZrNbTaMo HEAs under the dry wear condition (Hua et al., 2021).

Metal implants inevitably contact the surrounding tissue when implanted into the human body. Wear behaviour is unavoidable and must be considered for metal medical implants. Especially in bone implants, the high amount of frictional behaviour puts the material at risk of wear failure. Furthermore, implant wear may lead to inflammation and osteolysis, which affect implant longevity and increase the patient’s risk of secondary injury. Tribocorrosion includes the interaction of corrosion with sliding wear, biological solutions, solid particle erosion, frictional oxidation, cavitation erosion, abrasion, and fretting (Wood, 2007). Figures 7A–C shows two types of medical mental implants wear in the human body environment, namely, two-body wear and three-body wear. Compared with two-body wear, three-body wear has an additional interaction with particles that are dropped by wear, and two-body wear will eventually transform into three-body wear over time (Li et al., 2021).

Bhardwaj designed AlxTiZrNbHf (x = 0, 0.25, 0.50, 0.75, 1) bio-HEA to explore the effect of Al on the friction resistance. The reason why the addition of Al can enhance the wear resistance is because Al improves the mechanical properties of the material. Furthermore, an oxide film with higher friction resistance grows on the surface of AlTiZrNbHf due to the Al element, which is found without any elemental separation in the friction track in EDS analysis (Bhardwaj et al., 2021).

In fact, the friction and corrosion behaviour of metal implants often occur in combination. The corrosion behaviour may accelerate the wear situation of the material; conversely, the peeling of the oxide film due to wear may accelerate the corrosion of the material. TiZrHfNbFe bio-HEA exhibited good wear resistance in the dry friction test. Although corrosive wear occurs in a phosphate buffer saline (PBS) solution, the final performance is better than that of Ti6Al4V (Wang et al., 2022d). In addition, Hua (Hua et al., 2021) pointed out that the friction resistance of TiZrNbTaMo increases with decreasing Ti content. The wear is worse in the dry environment than in the PBS environment. The reason is that the oxide films formed in the dry environment are brittle and are more likely to fall off during friction, as shown in Figure 7D. The peeled oxide film changes the wear environment from two-body wear to three-body wear.

Surface modification is a common and effective method to improve bio-HEA surfaces and reduce friction loss. The film with a laminated structure of NbMoWTa has good friction resistance, especially when the film height is 2.5 nm; it exhibits excellent friction resistance, and the coefficient of friction (COF) is significantly lower than that of the monolithic NbMoWTa film (Luo et al., 2021).

## Conclusion

In recent years, many studies have aimed to determine the biomedical potential of HEAs to design excellent medical metal implants and expand the application range of bio-HEA. Compared with traditional medical metals, bio-HEAs have more freedom in composition selection and could be widely used in medical implants, especially in bone scaffolds, bone plates, and bone nails. The microstructure and morphology of bio-HEAs are closely affected by the selected elements or the proportion of each element. Strategies for designing reasonable and excellent HEA systems need to be further investigated.

In this review, the superior mechanical properties and biocompatibility of bio-HEAs are summarized. For mechanical properties, bio-HEAs could both have high yield strength and low modulus, which meet the strength requirements and avoid stress shielding. Furthermore, some bio-HEAs with superelasticity could be developed in the medical field. However, fatigue experiments are still lacking in bio-HEAs and need to be further evaluated. For biocompatibility, the elements selected by bio-HEAs focus on having excellent biocompatibility and low biotoxicity, for instance, Ti, Ta, Nb, Zr, and Hf. More importantly, the cytocompatibility of some bio-HEAs was even higher than that of CP-Ti and Ti6Al4V. However, the current cell viability tests of bio-HEAs focus on *in vitro* cell viability, lacking relevant *in vivo* animal experiments.

Although bio-HEAs have made significant progress, the availability of new bio-HEAs still has many properties to test. In addition, many medical properties of bio-HEAs have surpassed those of traditional medical metals. However, most of these studies only show a certain medical performance of bio-HEAs, and there is a lack of systematic and complete research on all the medical implant indices possessed by a certain material. In the future, one approach to obtain excellent bio-HEAs is by designing the bio-HEA composition and regulating the microstructure and morphology. The new bio-HEA is expected to become a new generation of metal medical implants with excellent performance.
